# Clinical application of HMGB1-TLR4 signaling pathway-mediated neuroinflammatory markers in infantile epileptic spasms syndrome

**DOI:** 10.3389/fimmu.2026.1801505

**Published:** 2026-05-01

**Authors:** Hui Chen, Jianmin Zhong, Yong Chen, Huaping Wu, Ruiyan Wang, Zhaoshi Yi, Xingying Zeng

**Affiliations:** 1 Department of Neurology, Children’s Hospital of Jiangxi Province, Nanchang, China; 2 Central Laboratory, Children’s Hospital of Jiangxi Province, Nanchang, China

**Keywords:** HMGB1, infantile epileptic spasms syndrome, neuroinflammatory, prednisone, TLR4

## Abstract

**Background:**

Infantile epileptic spasms syndrome (IESS) is a severe age-specific epileptic encephalopathy with unclear pathogenesis, and neuroinflammation is involved in its progression. The HMGB1-TLR4 signaling pathway, a key neuroinflammatory mediator in various epilepsies, has not been studied for its role and clinical biomarker potential in IESS.

**Methods:**

A retrospective study included 66 IESS patients treated with a modified prednisone regimen and 53 age-matched healthy controls. Serum HMGB1, TLR4, IL-1β, IL-2, IL-2R, IL-8 and TNF-α were detected by ELISA/chemiluminescent immunoassay in IESS patients (pre- and 2-week post-treatment) and controls; clinical data were collected via electronic medical records and follow-up.

**Results:**

IESS patients had significantly higher serum HMGB1, TLR4, IL-2, IL-2R, IL-8 and TNF-α than controls (*P* < 0.05; no IL-1β difference, *P*>0.05), and these elevated indicators decreased markedly post-treatment (*P* < 0.05). Logistic regression showed identified etiology and focal seizures were risk factors for short-term prednisone ineffectiveness, while Δ_Pre-Post_ HMGB1 was a protective factor (*P* < 0.05). Long-term follow-up (≥18 months) found identified etiology to be a risk factor for uncontrolled epilepsy and poor neurodevelopment (*P* < 0.05); early spasm remission and long-term seizure control were protective factors for neurodevelopment (*P* < 0.05).

**Conclusions:**

The HMGB1-TLR4 pathway mediates neuroinflammation in IESS pathogenesis and may serve as a therapeutic target. A high Δ_Pre-Post_ HMGB1 level was identified as a protective factor against short-term treatment failure of prednisone, indicating that dynamic monitoring of HMGB1 has clinical value for predicting short-term treatment responses to prednisone, though it does not predict long-term seizure control or neurodevelopmental outcomes. Identified etiology is a common risk factor for poor IESS outcomes, highlighting the importance of early etiological screening and sustained seizure control for IESS management.

## 1 Introduction

Infantile epileptic spasms syndrome (IESS) is a unique age-specific epileptic syndrome with an incidence of 2–5 cases per 10,000 live births. Onset typically occurs before 1 year of age, clinically characterized by head-nodding and embrace-like epileptic spasms, often accompanied by developmental arrest and/or regression. The condition poses substantial treatment challenges and carries a poor prognosis, imposing heavy burdens on affected children, their families, and society (1–8). Its etiologies are diverse, including genetic, metabolic, structural, immunological, and infectious factors that may act alone or synergistically, rendering IESS pathogenesis highly complex; notably, the underlying cause remains unidentified in ~35% of cases (9). Despite growing research efforts, the precise pathogenesis of IESS remains elusive due to its multifactorial etiology (9).

In recent years, neuroinflammation has emerged as a key research focus in epileptogenesis, particularly in refractory epilepsy, where impaired balance between activation and inactivation of inflammatory cells and mediators contributes critically to recurrent seizures (10, 11). As a distinct epileptic encephalopathy, IESS responds favorably to adrenocorticotropic hormone (ACTH) or glucocorticoid therapy (12–14), implying a potential role of neuroimmune or inflammatory responses in its pathogenesis. Previous studies have shown that serum levels of interleukin-2 (IL-2), tumor necrosis factor (TNF), and interferon (IFN) are significantly elevated in children with IESS compared to healthy controls, suggesting cytokine dysregulation may drive disease onset (15). Yamanaka et al. (16) reported that clinical and electroencephalogram (EEG) improvements in IESS patients after ACTH treatment were accompanied by marked increases in serum interleukin-1 receptor antagonist (an anticonvulsant), indicating a correlation between this cytokine and symptom amelioration. A recent flow cytometry-based study analyzed peripheral blood mononuclear cells (PBMCs) from 25 IESS patients and 54 age-matched healthy controls, identifying distinct immune dysregulation patterns (including reduced CD4+ T cell counts, altered CD4/CD8 ratios, and decreased TNF-α production in CD4+ T cells) and confirming immune-inflammatory imbalance in IESS (17). Our team has long focused on prednisone therapy for IESS, demonstrating that oral prednisone is effective and well-tolerated (12, 13). Our preliminary data further showed that pre-treatment serum levels of IL-2 receptor (IL-2R), IL-8, and TNF-α were significantly higher in IESS patients than in controls, and these levels decreased markedly following prednisone administration alongside clinical improvement (18). Collectively, these findings indicate neuroinflammation is involved in IESS pathogenesis. However, the mechanisms by which inflammatory dysregulation leads to IESS, and the key signaling pathways mediating this process, remain unclear. Identifying these pathways is crucial, as it will not only elucidate IESS pathogenesis but also facilitate the development of prognostic biomarkers and therapeutic targets.

High mobility group box 1 (HMGB1), a 215-amino-acid non-histone DNA-binding protein with high electrophoretic mobility due to its rich charged amino acid content, was first discovered in calf thymus by Goodwin and Johns in 1976 (19). As a highly conserved, widely expressed protein, HMGB1 regulates nucleosome stability, gene transcription, DNA replication, and repair (20, 21). Accumulating evidence has defined HMGB1 as a key mediator of systemic inflammation: it can be actively released by activated immune cells or passively secreted by necrotic/damaged cells following acetylation; extracellular HMGB1 is modified by reactive oxygen species to form a stable disulfide dimer, which binds to its receptors and triggers inflammation via nuclear factor-kappa B (NF-κB) signaling, leading to tissue damage (21, 22). HMGB1 interacts with multiple receptors, among which toll-like receptor 4 (TLR4), TLR2, and receptor for advanced glycation end products (RAGE) are the most critical. Notably, the HMGB1/TLR4 signaling pathway is closely linked to epilepsy. As an innate immune receptor widely expressed in the nervous system (neurons, microglia, astrocytes), TLR4 mediates macrophage activation, cytokine production, and tissue damage in response to bacterial infections and endogenous injuries (23). Maroso et al. (24) reported HMGB1/TLR4 pathway activation in both acute and chronic epilepsy models in adult mice, and blocking this axis reduced chronic seizure frequency and severity. Yang et al. (25) detected significantly elevated expression of HMGB1, TLR4, TNF-α, and IL-1β in hippocampal tissues from a rat model of mesial temporal lobe epilepsy (MTLE) and resected specimens from pediatric MTLE patients, confirming the pathway’s role in MTLE pathogenesis. Clinical studies have yielded consistent results: one study of 180 newly diagnosed epilepsy patients and 40 healthy children found that serum HMGB1 levels were significantly higher in the epilepsy group, and positively correlated with seizure frequency and epileptiform discharge index while negatively correlated with intelligence quotient, suggesting HMGB1 as a prognostic biomarker (26). Another study demonstrated a link between serum HMGB1 levels and inflammatory severity in epilepsy (27), with a growing body of related research published in recent years (28–30). Collectively, these findings identify HMGB1-TLR4-mediated neuroinflammation as a pathogenic mechanism in epilepsy.

Notably, despite extensive evidence linking the HMGB1-TLR4 pathway to non-IESS epilepsy, no studies have investigated this association in IESS. As a distinct epileptic syndrome characterized by unique spasms, EEG hypsarrhythmia, developmental delay, and responsiveness to hormonal therapy, IESS requires independent investigation of its relationship with the HMGB1-TLR4 pathway. Building on our previous findings of elevated serum IL-2R, IL-8, and TNF-α in IESS patients and the proven efficacy of ACTH/oral prednisone in treating IESS (12–14), we hypothesize that the HMGB1-TLR4 signaling pathway—a key node in the neuroinflammatory network—may contribute to IESS pathogenesis. This study detects the serum expression levels of key HMGB1-TLR4 signaling pathway proteins (HMGB1, TLR4) and related neuroinflammatory indicators (IL-1β, IL-2, IL-2R, IL-8, TNF-α) in children with IESS. It aims to preliminarily explore the role of HMGB1-TLR4-mediated neuroinflammation in IESS pathogenesis, clarify the clinical significance of these indicators and identify their potential as objective biomarkers, investigate their correlation with short-term prednisone efficacy (Efficacy after 2 weeks of prednisone treatment), and evaluate their predictive value for long-term seizure control and neurodevelopmental outcomes in patients with IESS. No relevant research has been reported to date.

## 2 Patients and methods

### 2.1 Study subjects

This was a retrospective study. We enrolled infants and young children with IESS who were treated in our hospital from May 2020 to June 2024, as well as healthy infants and young children who underwent physical examinations during the same period. Inclusion criteria for the case group (IESS group) (1): Meeting the diagnostic criteria for IESS defined by the International League Against Epilepsy (ILAE) (31): age at onset of 1–24 months, clinical presentation of epileptic spasms, ictal synchronous electroencephalogram (EEG) consistent with epileptic spasm patterns; interictal EEG showing hypsarrhythmia, multifocal or focal epileptiform discharges; may be accompanied by motor and intellectual developmental delay (early development may be normal) (2); Newly diagnosed IESS patients (3); Patients who received prednisone monotherapy as the first-line treatment (4); Patients with pre-stored serum samples (Note: Our department has focused on clinical research on prednisone treatment for IESS in recent years. Serum samples were routinely collected and stored before prednisone treatment and 2 weeks after treatment, with informed consent obtained from the patients’ families). Exclusion criteria (1): Patients with obvious cardiac, hepatic, or renal insufficiency (2); Patients who did not receive standardized prednisone treatment for IESS (3); Patients complicated with immune system diseases (4); Patients complicated with infections during prednisone treatment (5); Patients with incomplete important data, such as information on epileptic spasm seizures, cranial imaging, video EEG, and developmental quotient (DQ) assessed by the Gesell Development Scale.

All the research subjects participated in this study with the informed consent of their parents. This study was approved by the Medical Ethics Committee of Jiangxi Children’s Hospital(Ethical approval number:JXSETYY-YXKY-20250155). The control group consisted of healthy infants and young children who underwent physical examinations during the same period, for whom blood samples had been collected with parental consent in previous studies.

### 2.2 Treatment regimen

All enrolled IESS patients received prednisone treatment. The prednisone regimen was modified based on the protocol provided by Lux et al. (32), with specific details as follows (1): Prednisone 40 mg/d, divided into 4 doses, orally for 1 week. (a), If seizures persisted after 1 week, the dose was increased to 60 mg/d, divided into 4 doses, orally for 1 week; regardless of whether seizures were controlled, the dose was reduced to 40 mg/d, divided into 4 doses, orally for 1 week. (b), If seizures were absent after 1 week, no dose increase was required, and 40 mg/d (divided into 4 doses) was continued orally for 1 week (2); Dose reduction: 30 mg/d, once daily, orally in the morning for 1 week (3); Dose reduction: 20 mg/d, once daily, orally in the morning for 1 week (4); Dose reduction: 10 mg/d, once daily, orally in the morning for 1 week (5); Dose reduction: 5 mg/d, once daily, orally in the morning for 1 week (6); Dose reduction: 5 mg/d, once every other day, orally in the morning for 1 week (7); Discontinuation of medication. If seizures still occurred 2 weeks after prednisone treatment, physicians comprehensively considered the child’s condition and the family’s opinions to select other anti-seizure medications for treatment.

### 2.3 Index detection

Serum samples had been collected and stored according to the standard procedure previously (serum samples were routinely collected and stored from IESS patients before prednisone treatment and 2 weeks after treatment; for the control group, serum samples were collected once during the health check-up). The specific procedure and detection methods were as follows: 4 mL of venous blood was collected from each child, centrifuged at 3000 r/min for 10 minutes, and the serum was aliquoted and stored at -80°C until detection. Serum HMGB1 and TLR4 levels were detected by enzyme-linked immunosorbent assay (ELISA) using a Thermo MK3 microplate reader, with kits provided by Jiangsu Meimian Industrial Co., Ltd. (Jiangsu, China). Serum levels of IL-1β, IL-2,IL-2R, IL-8, and TNF-α were measured by chemiluminescence immunoassay using an IMMULITE 1000 chemiluminescence immunoanalyzer (Siemens AG, Germany), with kits provided by Siemens AG, Germany. All detection indicators were tested in accordance with the reagent instructions.

### 2.4 Collection and registration of historical data

Historical data were mainly collected and queried through the electronic medical record system. For data missing from the electronic medical record system, supplementary collection was performed through outpatient visits, WeChat, or telephone follow-up, followed by registration. The cutoff date for follow - up was set as December 2025. The collected data mainly included: general information of patients (age, age at onset), seizure characteristics (including seizure type, number of seizure clusters, seizure control after medication treatment, recurrence), developmental status [e.g., developmental quotient (DQ) assessed by the Gesell Development Scale], adverse drug reactions, results of liver and kidney function tests, blood routine, cranial imaging, and video EEG.

Definitions of terms related to IESS during historical data collection were as follows (1): Clinical remission of epileptic spasms was defined as no observation of epileptic spasms for at least 28 days, recorded by parents and caregivers in seizure diaries and confirmed by long-term video EEG monitoring (2). EEG remission was defined as no hypsarrhythmia on long-term video EEG for at least 28 days (3). IESS remission referred to remission of both epileptic spasms and EEG for at least 28 days (4). Recurrence was defined as the reoccurrence of IESS after achieving remission for at least 28 days. IESS recurrence was considered to meet one of the following criteria: a. Recurrence of epileptic spasms, other seizure types, or both; b. Reappearance of hypsarrhythmia on EEG. The definitions of the above terms were mainly based on previous definitions in the literature (33–35).

Additional notes:(1)Evaluation of DQ results: A normal DQ is defined as a score > 85; a borderline state is assigned to a DQ ranging from 76 to 85; mild developmental delay corresponds to a DQ of 55 to 75; moderate developmental delay is indicated by a DQ of 40 to 54; severe developmental delay is represented by a DQ of 25 to 39; and profound developmental delay is diagnosed when the DQ is < 25. Given that children with moderate developmental delay or milder conditions are likely to complete their education in regular schools or special schools for intellectual disabilities, the prognosis was classified as favorable for participants with normal development to moderate developmental delay, and unfavorable for those with severe or profound developmental delay (including cases of death).(2)Etiological classification of epilepsy: Based on the 2017 ILAE classification of epilepsy etiologies, the causes of epilepsy were categorized into structural, genetic, infectious, metabolic, immune, and unknown causes. For cases with overlapping etiologies, a hierarchical principle was applied (1): coexisting genetic and structural etiologies were classified as genetic, representing the fundamental molecular pathogenesis (2); coexisting genetic and metabolic, or metabolic and structural etiologies were classified as metabolic, as inborn errors of metabolism act as progressive and dominant drivers of encephalopathy.

### 2.5 Outcome measures

The primary outcome measures of this study included (1): Comparison of serum levels of HMGB1, TLR4, IL-1β, IL-2, IL-2R, IL-8, and TNF-α between the IESS group (baseline) and the healthy control group, as well as between pre- and post-2-week prednisone treatment in the IESS group (2); Correlation between biomarkers, pre-treatment versus 2-week post-prednisone treatment changes, and short-term efficacy of prednisone in the IESS group(Efficacy after 2 weeks of prednisone treatment); For each biomarker, the pre-treatment minus 2-week post-prednisone treatment change value (Δ_Pre−Post_) was calculated using the formula: Δ_Pre−Post_=C_pre-treatment_−C_2w-post-treatment_, where C_pre-treatment_ represents the baseline concentration before prednisone administration and C_2w-post-treatment_ represents the concentration after 2 consecutive weeks of treatment (3). Predictive value of neuroinflammatory indicators for long-term seizure control in patients with IESS during follow-up. Long-term seizure control was defined as the absence of any epileptic seizures during the 28 days before the final follow-up (36) (4). Predictive value of neuroinflammatory indicators for neurodevelopmental outcomes at the end of follow-up.

### 2.6 Statistical analysis

Statistical analysis of the data was performed using SPSS 25.0 software. Measurement data with a normal distribution were expressed as mean ± standard deviation ( $\bar{x}$ ± s), and comparisons between the two groups were conducted using the t-test. Measurement data with a non-normal distribution were presented as median (interquartile range) M (P25, P75), and intergroup comparisons were carried out using the Mann-Whitney U test. Categorical data were expressed as numbers and percentages n (%), and comparisons of categorical data were made using the chi-square test or Fisher’s exact test. Logistic regression analysis was employed to identify risk factors. *P* value < 0.05 was considered statistically significant.

## 3 Results

### 3.1 Comparisons of baseline data between the IESS group and control group, and of indicators before and after treatment in the IESS group

**Figure 1:**
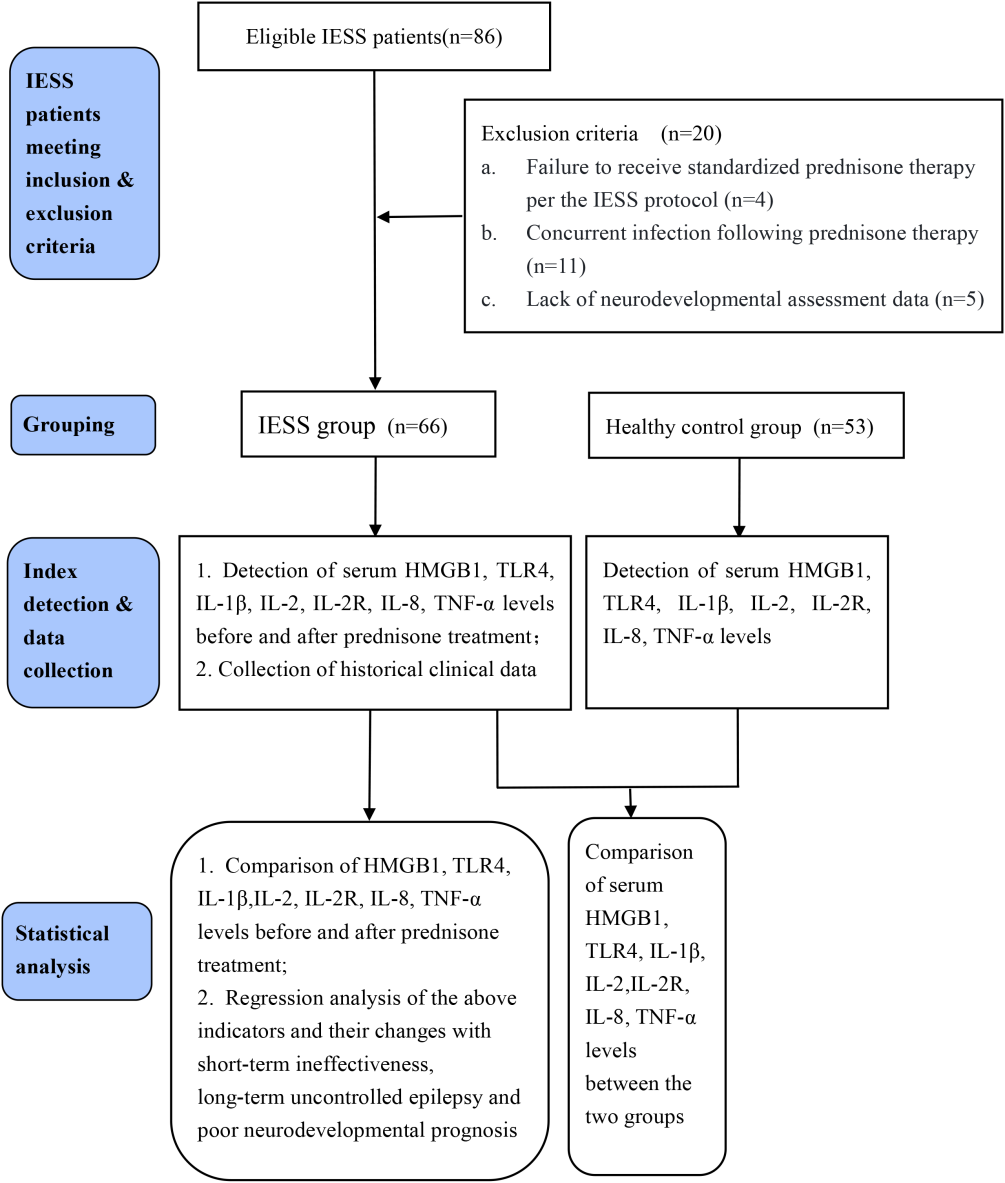
Study flow chart.

A retrospective data extraction and analysis identified 86 children with IESS who met the inclusion criteria, among whom 20 were excluded after screening against the exclusion criteria, resulting in a final cohort of 66 children with IESS enrolled in this study. The exclusion reasons were as follows (1): non-standard prednisone therapy for IESS (n=4) (2); infection complicated after prednisone treatment (n=11) (3); incomplete neurodevelopmental assessment data (n=5). A total of 53 healthy infants who underwent physical examination during the same period were recruited as the control group (**Figure 1**).

In the IESS group (n=66), 46 were male (69.7%) and 20 were female (30.3%); in the healthy control group (n=53), 32 were male (60.4%) and 21 were female (39.6%). The median (interquartile range) ages were 7 (5, 11) months in the IESS group and 6 (5, 7) months in the control group. No statistically significant differences were observed in gender and age between the two groups (*P*>0.05). Serum levels of HMGB1, TLR4, IL-2, IL-2R, IL-8 and TNF-α in the IESS group were significantly higher than those in the control group (all *P* < 0.05), while no significant difference was found in serum IL-1β level between the two groups (*P*>0.05) (**Table 1**). After 2 weeks of prednisone treatment, serum levels of HMGB1, TLR4, IL-2, IL-2R, IL-8 and TNF-α in the IESS group were significantly lower than those before treatment (all *P* < 0.05), and there was no significant change in serum IL-1β level before and after treatment (*P*>0.05) (**Table 2**).

**Table 1:** Comparison of baseline data between the IESS group and control group.

Index	IESS group (n=66)	Control group (n=53)	Test statistic	*P* value
Male [n (%)]	46(69.7)	32(60.4)	*χ*^2^ = 1.131	0.288
Age (months)	7 (5, 11)	6(5,7)	*Z* = -1.799	0.072
HMGB1(pg/mL)	1109.44 ± 744.49	460.81 ± 175.65	*t* = 6.845	0.000
TLR4(ng/mL)	1.16 ± 0.64	0.87 ± 0.46	*t* = -2.880	0.005
IL-1β(pg/mL)	3.39 ± 1.24	3.27 ± 1.32	*t* = 0.537	0.592
IL-2(pg/mL)	7.40 ± 4.02	3.50 ± 2.14	*t* = 6.772	0.000
IL-2R(U/mL)	1130.44 ± 409.59	734.99 ± 509.58	*t* = -4.584	0.000
IL-8(pg/mL)	115.52 ± 100.75	64.97 ± 45.55	*t* = -3.639	0.000
TNF-α(pg/mL)	13.63 ± 7.98	10.93 ± 5.40	*t* = -2.196	0.030

n, number of cases.

**Table 2:** Comparison of data in the IESS group before and after prednisone therapy.

Index	Pre-prednisone therapy (n=66)	Post-prednisone therapy (n=66)	Test statistic	*P* value
HMGB1(pg/mL)	1109.44 ± 744.49	726.74 ± 621.90	*t* = 5.166	0.000
TLR4(ng/mL)	1.16 ± 0.64	0.69 ± 0.30	*t* = 6.281	0.000
IL-1β(pg/mL)	3.39 ± 1.24	3.12 ± 1.62	*t* = 1.340	0.185
IL-2(pg/mL)	7.40 ± 4.02	4.28 ± 2.14	*t* = 5.979	0.000
IL-2R(U/mL)	1130.44 ± 409.59	580.05 ± 213.17	*t* = 10.125	0.000
IL-8(pg/mL)	115.52 ± 100.75	56.53 ± 34.74	*t* = 4.828	0.000
TNF-α(pg/mL)	13.63 ± 7.98	8.47 ± 5.40	*t* = 4.313	0.000

n, number of cases.

### 3.2 Short-term efficacy of prednisone therapy for IESS and analysis of influencing factors

Among all 66 children with IESS, the median age at onset was 6 (4, 7) months. A total of 43 cases (65.2%) had a disease course of <2 months before treatment and 23 cases (34.8%) had a course of ≥2 months; 34 cases (51.5%) had identified etiologies (including 13 genetic, 18 structural and 3 metabolic etiologies), and 32 cases (48.5%) were of unknown etiology. Before prednisone therapy, 18 cases (27.3%) had concomitant focal seizures, and 48 cases (72.7%) presented with neurodevelopmental abnormalities (DQ ≤75) prior to onset; electroencephalography (EEG) showed typical hypsarrhythmia in 26 cases (39.4%) and variant hypsarrhythmia in 40 cases (60.6%). At 2 weeks after the initiation of prednisone therapy (with seizure-freedom achieved at ≤2 weeks of treatment and confirmed during subsequent follow-up), 45 cases (68.2%) achieved clinical remission of epileptic spasms, 43 cases (65.2%) had EEG remission, and 39 cases (59.1%) achieved complete IESS remission (concomitant clinical and EEG remission). Additionally, 6 cases achieved complete IESS remission after 2 weeks of prednisone therapy, all of whom received adjuvant ASMs.

Adverse events were observed in 86 children with IESS who received prednisone therapy (including the 66 enrolled cases and 20 excluded cases who underwent prednisone treatment): Cushingoid features (e.g., moon face, central obesity) in 70 cases, increased appetite in 71 cases, irritability in 21 cases, complicated infection in 11 cases, somnolence in 6 cases, and hypertension in 3 cases. Cushingoid features and increased appetite resolved following dose reduction and discontinuation of prednisone, while irritability and somnolence mostly occurred in the early stage of treatment and alleviated or resolved with prolonged therapy. Transient hypertension was noted in 3 cases without accompanying irritability or crying, and normalized spontaneously without specific intervention. No severe adverse events including hepatic or renal dysfunction, adrenocortical insufficiency or crisis, or mortality were observed in any child during prednisone therapy.

Univariate logistic regression analysis revealed that identified etiology and concomitant focal seizures were significant risk factors for short-term treatment failure of prednisone therapy for IESS (all *P* < 0.05), while a high pre-post treatment change in HMGB1 level (Δ_Pre-Post_ HMGB1) was a significant protective factor (*P* < 0.05); no other indicators showed statistical significance (all *P*>0.05) (**Table 3**). Multivariate logistic regression analysis incorporating the statistically significant factors confirmed that identified etiology, concomitant focal seizures and elevated Δ_Pre-Post_ HMGB1 level remained independent significant factors (all *P* < 0.05) (**Table 4**).

**Table 3:** Univariate logistic regression analysis of risk factors for short-term ineffectiveness of initial prednisone therapy in IESS.

Index	Index value	OR value	95% CI	*P* value
Male [n (%)]	46 (69.7)	0.786	0.272-2.271	0.656
Onset age (months)	6 (4,7)	0.876	0.743-1.032	0.114
Disease course before therapy < 2 months [n (%)]	43 (65.2)	1.547	0.555-4.314	0.404
IESS with identified etiology [n (%)]	34 (51.5)	7.000	2.272-21.569	0.001
Concurrent focal seizures [n (%)]	18 (27.3)	6.314	1.893-21.062	0.003
Abnormal neurodevelopment before onset [n (%)]	48 (72.7)	1.122	0.371-3.399	0.838
Proportion of variant hypsarrhythmia [n (%)]	40 (60.6)	2.714	0.935-7.877	0.066
Baseline HMGB1(pg/mL)	1109.44 ± 744.49	1.000	0.999-1.001	0.899
Baseline TLR4(ng/mL)	1.16 ± 0.64	1.006	0.462-2.190	0.987
Baseline IL-1β(pg/mL)	3.39 ± 1.24	1.145	0.768-1.707	0.507
Baseline IL-2(pg/mL)	7.40 ± 4.02	0.974	0.861-1.103	0.679
Baseline IL-2R(U/mL)	1130.44 ± 409.59	1.000	0.999-1.001	0.899
Baseline IL-8(pg/mL)	115.52 ± 100.75	1.003	0.999-1.008	0.171
Baseline TNF-α(pg/mL)	13.63 ± 7.98	1.035	0.972-1.101	0.288
Δ_Pre-Post_ HMGB1(pg/mL)	382.70 ± 601.86	0.996	0.994-0.998	0.000
Δ_Pre-Post_ TLR4(ng/mL)	0.46 ± 0.60	1.545	0.669-3.570	0.308
Δ_Pre-Post_ IL-1β(pg/mL)	0.27 ± 1.64	1.055	0.779-1.428	0.730
Δ_Pre-Post_ IL-2(pg/mL)	3.12 ± 4.24	0.992	0.883-1.115	0.896
Δ_Pre-Post_ IL-2R(U/mL)	550.39 ± 441.61	1.000	0.999-1.001	0.592
Δ_Pre-Post_ IL-8(pg/mL)	58.98 ± 99.25	1.004	0.999-1.010	0.098
Δ_Pre-Post_ TNF-α(pg/mL)	5.16 ± 9.73	1.027	0.975-1.082	0.320

*n*, number of cases; male, disease course < 2 months before therapy, identified etiology, concurrent focal seizures, abnormal neurodevelopment before onset, and variant hypsarrhythmia were each assigned a value of 1; ΔPre-Post, C_pre-therapy_−C_2-week therapy_, where C_pre-therapy_ is the baseline concentration of each indicator before prednisone administration, and C_2-week therapy_ is the corresponding concentration after 2 consecutive weeks of prednisone therapy; OR, odds ratio; CI, confidence interval.

**Table 4:** Multivariate logistic regression analysis of risk factors for short-term ineffectiveness of prednisone therapy, long-term uncontrolled epilepsy and poor neurodevelopmental prognosis in IESS.

Index	OR value	95% CI	*P* value
Short-term ineffectiveness of prednisone therapy			
IESS with identified etiology	5.949	1.280-27.647	0.023
Concurrent focal seizures	9.709	1.365-69.081	0.023
ΔPre-Post HMGB1(pg/mL)	0.996	0.994-0.998	0.001
Long-term uncontrolled epilepsy			
IESS with identified etiology	13.437	3.622-49.853	0.000
IESS remission within 2 weeks of prednisone therapy	0.907	0.236-3.480	0.887
Poor neurodevelopmental prognosis in IESS			
IESS with identified etiology	5.360	1.276-22.523	0.022
IESS remission within 2 weeks of prednisone therapy	0.215	0.051-0.912	0.037
Seizure control at the last follow-up	0.174	0.040-0.761	0.020
Δ_Pre-Post_ IL-8(pg/mL)	1.006	0.998-1.014	0.129

*n*, number of cases; identified etiology, concurrent focal seizures, IESS remission within 2 weeks of prednisone therapy, and seizure control at the last follow-up were each assigned a value of 1; ΔPre-Post, C_pre-therapy_−C_2-week therapy_, where C_pre-therapy_ is the baseline concentration of each indicator before prednisone administration, and C_2-week therapy_ is the corresponding concentration after 2 consecutive weeks of prednisone therapy; OR, odds ratio; CI, confidence interval.

### 3.3 Risk factor analysis for long-term uncontrolled epilepsy in IESS

A long-term follow-up was conducted in this study, with the shortest follow-up duration of 18 months and the longest of 67 months. Among the 45 children who achieved IESS remission (including those with a therapeutic response within and after 2 weeks of treatment), 18 cases (40.0%) experienced disease recurrence during follow-up. At the last follow-up, 30 of the 66 children with IESS (45.5%) achieved sustained seizure control, while 36 cases (54.5%) had uncontrolled epilepsy. This latter group included 2 patients who died of status epilepticus, both of whom had discontinued prednisone therapy for six months prior to death.

Univariate logistic regression analysis demonstrated that an identified etiology was a significant risk factor for long-term uncontrolled epilepsy in IESS (*P* < 0.05), whereas IESS remission achieved within 2 weeks of prednisone initiation served as a significant protective factor (*P* < 0.05). No other indicators were found to have statistical significance (all *P*>0.05) (**Table 5**). However, when the statistically significant factors were incorporated into the multivariate logistic regression analysis, only an identified etiology remained a statistically significant factor (*P* < 0.05), and the protective effect of IESS remission within 2 weeks of prednisone therapy was not statistically significant (*P*>0.05) (**Table 4**).

**Table 5:** Univariate logistic regression analysis of risk factors for long-term uncontrolled epilepsy in IESS.

Index	Index value	OR value	95% CI	*P* value
Male [n (%)]	46 (69.7)	1.300	0.454-3.725	0.625
Onset age (months)	6(4,7)	1.040	0.899-1.203	0.600
Disease course before therapy < 2 months [n (%)]	43(65.2)	1.485	0.531-4.155	0.451
IESS with identified etiology [n (%)]	34(51.5)	14.000	4.256-46.050	0.000
Concurrent focal seizures [n (%)]	18(27.3)	2.000	0.645-6.201	0.230
Abnormal neurodevelopment before onset [n (%)]	48(72.7)	1.286	0.434-3.808	0.650
Proportion of variant hypsarrhythmia [n (%)]	40(60.6)	1.048	0.389-2.823	0.927
IESS remission within 2 weeks of prednisone therapy [n (%)]	39(59.1%)	0.325	0.115-0.921	0.034
Proportion of patients with >2 ASMs at the last follow-up [n (%)]	37(56.1%)	1.571	0.590-4.189	0.366
Baseline HMGB1(pg/mL)	1109.44 ± 744.49	1.000	1.000-1.001	0.405
Baseline TLR4(ng/mL)	1.16 ± 0.64	2.029	0.873-4.715	0.100
Baseline IL-1β(pg/mL)	3.39 ± 1.24	0.729	0.481-1.107	0.138
Baseline IL-2(pg/mL)	7.40 ± 4.02	1.028	0.909-1.161	0.663
Baseline IL-2R(U/mL)	1130.44 ± 409.59	1.000	0.999-1.001	0.821
Baseline IL-8(pg/mL)	115.52 ± 100.75	1.003	0.997-1.008	0.330
Baseline TNF-α(pg/mL)	13.63 ± 7.98	0.958	0.900-1.020	0.182
Δ_Pre-Post_ HMGB1(pg/mL)	382.70 ± 601.86	1.000	0.999-1.001	0.941
Δ_Pre-Post_ TLR4(ng/mL)	0.46 ± 0.60	1.915	0.807-4.547	0.141
Δ_Pre-Post_ IL-1β(pg/mL)	0.27 ± 1.64	0.878	0.649-1.188	0.398
Δ_Pre-Post_ IL-2(pg/mL)	3.12 ± 4.24	0.991	0.883-1.112	0.879
Δ_Pre-Post_ IL-2R(U/mL)	550.39 ± 441.61	1.000	0.999-1.001	0.624
Δ_Pre-Post_ IL-8(pg/mL)	58.98 ± 99.25	1.005	1.000-1.011	0.065
Δ_Pre-Post_ TNF-α(pg/mL)	5.16 ± 9.73	0.971	0.922-1.023	0.269

*n*, number of cases; male, disease course < 2 months before therapy, identified etiology, concurrent focal seizures, abnormal neurodevelopment before onset, variant hypsarrhythmia, IESS remission within 2 weeks of prednisone therapy, and >2 ASMs at the last follow-up were each assigned a value of 1; ΔPre-Post, C_pre-therapy_ − C_2-week therapy_, where C_pre-therapy_ is the baseline concentration of each indicator before prednisone administration, and C_2-week therapy_ is the corresponding concentration after 2 consecutive weeks of prednisone therapy; OR, odds ratio; CI, confidence interval.

### 3.4 Risk factor analysis for poor long-term neurodevelopmental outcome in IESS

Following long-term follow-up (≥18 months), 32 cases (48.5%) achieved a favorable long-term neurodevelopmental outcome and 34 cases (51.5%) had a poor outcome. Univariate logistic regression analysis identified an identified etiology of IESS and elevated pre-post treatment change in IL-8 level (Δ_Pre-Post_ IL-8) as significant risk factors for poor long-term neurodevelopmental outcome in IESS (all *P* < 0.05). In contrast, IESS remission achieved within 2 weeks of prednisone initiation and sustained seizure control at the last follow-up were confirmed as significant protective factors against poor neurodevelopmental outcome (all *P* < 0.05). No other indicators exhibited statistical significance (all *P*>0.05) (**Table 6**). When these statistically significant factors were included in the multivariate logistic regression analysis, an identified etiology of IESS, IESS remission within 2 weeks of prednisone therapy and sustained seizure control at the last follow-up remained statistically significant (all *P* < 0.05), whereas the association between elevated Δ_Pre-Post_ IL-8 and poor neurodevelopmental outcome lost statistical significance (*P*>0.05) (**Table 4**). For all variables included in the above multivariate logistic regression analyzes, the tolerance values were significantly greater than 0.1 and the variance inflation factors (VIF) were all less than 10, indicating the absence of severe multicollinearity among the variables.

**Table 6:** Univariate logistic regression analysis of risk factors for poor long-term neurodevelopmental prognosis in IESS.

Index	Index value	OR value	95% CI	*P* value
Male [n (%)]	46 (69.7)	1.333	0.465-3.825	0.593
Onset age (months)	6(4,7)	0.911	0.784-1.058	0.222
Disease course before therapy < 2 months [n (%)]	43(65.2)	1.143	0.415-3.148	0.796
IESS with identified etiology [n (%)]	34(51.5)	16.714	4.954-56.397	0.000
Concurrent focal seizures [n (%)]	18(27.3)	1.359	0.457-4.035	0.581
Abnormal neurodevelopment before onset [n (%)]	48(72.7)	0.736	0.248-2.186	0.581
Proportion of variant hypsarrhythmia [n (%)]	40(60.6)	1.000	0.373-2.684	1.000
IESS remission within 2 weeks of prednisone therapy [n (%)]	39(59.1%)	0.127	0.041-0.395	0.000
Proportion of patients with >2 ASMs at the last follow-up [n (%)]	37(56.1%)	1.448	0.545-3.845	0.458
Seizure control at last follow-up [n (%)]	30(45.5%)	0.083	0.026-0.269	0.000
Baseline HMGB1(pg/mL)	1109.44 ± 744.49	1.000	1.000-1.001	0.227
Baseline TLR4(ng/mL)	1.16 ± 0.64	2.017	0.883-4.607	0.096
Baseline IL-1β(pg/mL)	3.39 ± 1.24	0.837	0.559-1.252	0.387
Baseline IL-2(pg/mL)	7.40 ± 4.02	1.065	0.942-1.204	0.315
Baseline IL-2R(U/mL)	1130.44 ± 409.59	1.001	0.999-1.002	0.413
Baseline IL-8(pg/mL)	115.52 ± 100.75	1.004	0.999-1.010	0.107
Baseline TNF-α(pg/mL)	13.63 ± 7.98	0.990	0.931-1.052	0.744
Δ_Pre-Post_ HMGB1(pg/mL)	382.70 ± 601.86	1.000	0.999-1.001	0.709
Δ_Pre-Post_ TLR4(ng/mL)	0.46 ± 0.60	1.649	0.714-3.812	0.242
Δ_Pre-Post_ IL-1β(pg/mL)	0.27 ± 1.64	0.902	0.669-1.217	0.500
Δ_Pre-Post_ IL-2(pg/mL)	3.12 ± 4.24	1.041	0.927-1.168	0.499
Δ_Pre-Post_ IL-2R(U/mL)	550.39 ± 441.61	1.000	0.999-1.001	0.499
Δ_Pre-Post_ IL-8(pg/mL)	58.98 ± 99.25	1.007	1.001-1.014	0.015
Δ_Pre-Post_ TNF-α(pg/mL)	5.16 ± 9.73	0.983	0.934-1.033	0.494

*n*, number of cases; male, disease course < 2 months before therapy, identified etiology, concurrent focal seizures, abnormal neurodevelopment before onset, variant hypsarrhythmia, IESS remission within 2 weeks of prednisone therapy, >2 ASMs at the last follow-up, and seizure control at the last follow-up were each assigned a value of 1; Δ_Pre-Post_, C_pre-therapy_ − C_2-week therapy_, where C_pre-therapy_ is the baseline concentration of each indicator before prednisone administration, and C_2-week therapy_ is the corresponding concentration after 2 consecutive weeks of prednisone therapy; OR, odds ratio; CI, confidence interval.

## 4 Discussion

IESS is a severe epileptic encephalopathy characterized by intractable epileptic spasms and neurodevelopmental arrest or regression, with neuroinflammation recognized as one of its key pathogenic drivers (37–39). This retrospective study investigated the role of the HMGB1-TLR4 signaling pathway—a critical mediator of neuroinflammatory responses—in IESS, and analyzed the correlation between related indicators and the short-term efficacy of prednisone (efficacy at 2 weeks post-treatment). We also explored whether neuroinflammatory markers could predict long-term seizure control and neurodevelopmental outcomes in IESS, aiming to identify potential biomarkers for the disease.

A previous study enrolled 180 children with new-onset epilepsy (including 67 with generalized tonic-clonic seizures, 92 with focal motor seizures, and 21 with IESS) and 40 healthy children, demonstrating that serum HMGB1 and IL-1β concentrations within 24 hours post-seizure were significantly higher in the epilepsy group than in the control group, with the most prominent elevation observed in children with IESS (26). In the present study, we extended the analysis to include not only HMGB1 and IL-1β but also TLR4, IL-2, IL-2R, IL-8, and TNF-α. Our results showed that serum levels of HMGB1, TLR4, and downstream proinflammatory cytokines (IL-2, IL-2R, IL-8, TNF-α) were significantly elevated in children with IESS compared with age-matched healthy controls, consistent with previous findings highlighting aberrant immune activation in IESS (18, 26). HMGB1 is defined as a key cytokine and a core protein in the inflammatory cellular network; it triggers the release of proinflammatory cytokines by activating its receptor TLR4 (23, 40), thereby inducing neuronal damage and excitotoxicity and promoting epileptogenesis. The upregulation of the HMGB1-TLR4 pathway observed in our study suggests that this pathway may be a pivotal driver of neuroinflammatory processes in IESS, providing a novel perspective for elucidating the disease’s pathogenesis. Targeting the HMGB1-TLR4 signaling axis may therefore represent a potential strategy for intervening in the immunopathological processes of IESS, though further research is required to validate this hypothesis. However, the levels of the measured markers in the patient group showed wide individual variation and obvious overlap with those in the control group, even though statistically significant differences were observed between groups. This phenomenon may be explained by the fact that IESS is a highly heterogeneous epileptic encephalopathy in early life. Patients vary substantially in etiology, age at onset, seizure frequency, spasm duration, and underlying brain developmental status. These clinical differences directly contribute to marked individual variability in the intensity of neuroinflammation and biomarker release, leading to a broad distribution of marker levels. Nevertheless, our findings still support the notion that HMGB1-TLR4-mediated neuroinflammation plays an important role in the pathogenesis of IESS.

Notably, we found that serum levels of HMGB1, TLR4, and proinflammatory cytokines (IL-2, IL-2R, IL-8, TNF-α) were significantly reduced at 2 weeks after prednisone treatment in children with IESS compared with baseline. Additionally, a high pre-post treatment change in HMGB1 level (Δ_Pre-Post_ HMGB1) was identified as a protective factor against short-term treatment failure of prednisone for IESS—i.e., a greater reduction in HMGB1 at 2 weeks post-treatment correlated with a higher probability of short-term remission. These findings indicate that prednisone, as a first-line immunomodulatory agent for IESS, exerts its anti-inflammatory effects by inhibiting the HMGB1-TLR4 signaling pathway and the synthesis of proinflammatory cytokines, thereby alleviating epileptic spasms; HMGB1 may thus serve as a key therapeutic target of prednisone in IESS. Previous research on Kawasaki disease has also demonstrated that glucocorticoids exert anti-inflammatory effects by inhibiting HMGB1: treatment of human coronary artery endothelial cells activated by serum from patients with Kawasaki disease with prednisolone was shown to suppress the extracellular release of HMGB1, downregulate the ERK1/2, JNK, p38, and NF-κB signaling pathways, and reduce the production of IL-1β and TNF-α (41). This study highlighted the critical role of extracellular HMGB1 in mediating the pathogenesis of Kawasaki disease and suggested that prednisolone treatment during the acute phase may ameliorate HMGB1-mediated inflammatory responses in Kawasaki vasculitis (41). Furthermore, our study revealed no correlation between the short-term remission of IESS with prednisone (2 weeks) and baseline serum levels of HMGB1, TLR4, IL-2, IL-2R, IL-8, or TNF-α; instead, remission was correlated with the pre-post treatment change in HMGB1. This finding indicates that the magnitude of HMGB1 reduction after treatment may serve as a predictive biomarker for short-term treatment outcomes, with important clinical implications: dynamic monitoring of HMGB1 levels at an early stage of treatment may enable the identification of patients with poor treatment responses, facilitating timely adjustments to individualized therapy (e.g., early add-on of other ASMs). It is important to note, however, that short-term treatment response to prednisone was associated not only with the objective indicator Δ_Pre-Post_ HMGB1 but also with other clinical factors. We identified an identified etiology of IESS and concomitant focal seizures as risk factors for short-term treatment failure of prednisone, emphasizing the need for clinicians to conduct a comprehensive assessment of multiple clinical factors when evaluating treatment responses.

A previous prospective clinical trial followed 65 children with IESS for 4 years, reporting that 37 (57%) continued to experience seizures and 28 (43%) achieved seizure freedom; among the 37 children with persistent seizures, 32.4% still had epileptic spasms (42). This trial also found that seizure control at day 14 did not influence seizure outcomes at 4 years, indicating suboptimal long-term seizure control in IESS. To further characterize long-term seizure control and identify its risk factors in IESS, we conducted an extended follow-up (≥18 months) in the present study. At the last follow-up, 30 of the 66 children with IESS (45.5%) achieved sustained seizure control, while 36 (54.5%) had uncontrolled epilepsy. Regression analyzes revealed that an identified etiology of IESS was a risk factor for long-term uncontrolled epilepsy, and that IESS remission within 2 weeks of prednisone treatment was not a protective factor against long-term uncontrolled epilepsy—findings consistent with the aforementioned study (42). None of the investigated inflammatory factors (HMGB1, TLR4, IL-1β, IL-2, IL-2R, IL-8, TNF-α) or their pre-post treatment changes were associated with long-term seizure control. The poor long-term seizure outcomes and the lack of correlation between inflammatory markers and long-term seizure control observed in our study may be attributed to two main factors. First, the etiology and pathogenesis of IESS are likely multifactorial, encompassing (39) (1): alterations in genetic and epigenetic regulation (2); stress responses and hypothalamic-pituitary-adrenal axis activation (3); neuroinflammation and immune dysfunction (4); aberrant neuronal transmission and signaling pathways; and (5) metabolic pathway dysfunction. These processes likely interact synergistically rather than acting independently, and neuroinflammation is only one component of the complex pathogenic cascade of IESS. This may explain why neuroinflammatory markers failed to predict long-term seizure control and why long-term seizure outcomes are suboptimal in IESS, suggesting that multi-targeted pharmacotherapy or combination therapy may be required for long-term seizure control and prognosis improvement in this population. Second, the prednisone regimen used in the present study was not a long-term maintenance therapy, with a total course of only 7–8 weeks; thus, we only analyzed the correlation between short-term pre-post treatment changes in inflammatory markers and long-term efficacy. Although prednisone exhibited significant short-term efficacy, the recurrence rate after discontinuation was high (40.0%), potentially due to the reactivation of inflammatory responses following drug withdrawal and subsequent spasm recurrence. Future studies should conduct long-term dynamic monitoring of neuroinflammatory markers to further elucidate their correlation with long-term seizure control and outcomes in IESS. Notably, a recent multicenter retrospective case series investigated a long-term ACTH regimen for IESS, consisting of sequential daily injections for a median of 16 days (range: 11–28 days) and weekly injections for a median of 10 months (range: 3–22 months) (43). This regimen achieved a 60.6% seizure-free rate at 24 months with no reported fatal adverse events, leading the authors to suggest that long-term ACTH therapy may be a treatment option for patients with recurrent or refractory IESS (43). However, further randomized controlled trials are needed to validate the efficacy of long-term glucocorticoid maintenance therapy for IESS.

To further explore the correlation between neuroinflammatory markers and long-term neurodevelopmental outcomes, we performed a risk factor analysis for poor long-term neurodevelopmental outcome in IESS. After long-term follow-up (≥18 months), 32 children (48.5%) achieved a favorable neurodevelopmental outcome, and 34 (51.5%) had a poor outcome. Regression analyzes identified an identified etiology of IESS as a risk factor for poor long-term neurodevelopmental outcome, while IESS remission within 2 weeks of prednisone treatment and sustained seizure control at the last follow-up were protective factors against poor neurodevelopmental outcomes. No correlation was observed between neuroinflammatory markers mediated by the HMGB1-TLR4 pathway and long-term neurodevelopmental outcomes. Previous studies have also indicated that the underlying etiology of IESS and early seizure remission after glucocorticoid treatment are associated with long-term prognosis (8, 44, 45), underscoring the importance of etiological diagnosis, early achievement of seizure remission, and prevention of recurrence in IESS. Given that the pre-post treatment reduction in HMGB1 serves as a predictive biomarker for short-term treatment outcomes, dynamic monitoring of HMGB1 levels at an early stage is warranted in clinical practice to identify patients with poor treatment responses and guide individualized therapy adjustments.

This study has several limitations that should be acknowledged. First, the retrospective study design may have introduced selection bias, and the relatively limited sample size may compromise the generalizability of our findings. Second, as an observational study, this research cannot establish a causal relationship between the HMGB1-TLR4 pathway and the pathogenesis of IESS; future interventional studies using animal models are required to validate this relationship more thoroughly. In fact, our team has conducted parallel animal experiments using a rat model of IESS established by prenatal stress combined with NMDA exposure. In these animal studies, we evaluated the effects of anti-HMGB1 neutralizing antibody and ACTH on seizure severity and neuroinflammation. We found that HMGB1, TLR4, proinflammatory cytokines (including IL-1β, IL-2R, IL-8, and TNF-α), and iNOS were significantly upregulated in brain tissue of IESS rat, whereas Arg1 was downregulated. Intervention with ACTH and anti-HMGB1 effectively suppressed the HMGB1-TLR4 pathway and reduced neuroinflammation, with combined treatment showing the best efficacy. Detailed results from these animal experiments will be reported separately in a dedicated mechanistic study. Regarding IL-1β, the cytokine is well established in epilepsy research. In our clinical cohort, peripheral IL-1β levels did not reach statistical significance, which may be attributed to the use of peripheral blood rather than central nervous system tissue. Notably, our parallel animal experiments revealed significantly higher IL-1β levels in brain tissue homogenates of IESS model rats compared with controls. This discrepancy may be related to the inherent differences between central and peripheral inflammatory profiles. Third, we only analyzed short-term pre-post treatment changes in inflammatory markers and did not conduct long-term dynamic monitoring of these indicators. Future studies should address this gap to further clarify the correlation between long-term neuroinflammatory marker dynamics and long-term seizure control and outcomes in IESS.

## 5 Conclusion

Despite these limitations, this study is the first to evaluate the role of the HMGB1-TLR4 signaling pathway in IESS and to preliminarily explore the neuroinflammatory mechanisms mediated by this pathway in the disease, suggesting that targeting the HMGB1-TLR4 pathway may represent a novel therapeutic strategy for IESS. A high Δ_Pre-Post_ HMGB1 level was identified as a protective factor against short-term treatment failure of prednisone, indicating that dynamic monitoring of HMGB1 has clinical value for predicting short-term treatment responses to prednisone, though it does not predict long-term seizure control or neurodevelopmental outcomes. An identified etiology of IESS was a consistent risk factor for short-term treatment failure, long-term uncontrolled epilepsy, and poor long-term neurodevelopmental outcome, while IESS remission within 2 weeks of prednisone treatment and sustained seizure control at the last follow-up were protective factors against poor long-term neurodevelopmental outcomes. These findings emphasize the clinical importance of etiological diagnosis, early achievement of seizure remission, and prevention of recurrence in IESS. Future prospective studies with larger sample sizes are needed to validate our results, and mechanistic studies (e.g., using IESS animal models) are required to establish a causal relationship between the HMGB1-TLR4 pathway and the development of IESS—ultimately translating these findings into improved clinical management and outcomes for children with this devastating disease.

## Data Availability

The raw data supporting the conclusions of this article will be made available by the authors, without undue reservation.
